# Diet-Related Factors, Physical Activity, and Weight Status in Polish Adults

**DOI:** 10.3390/nu11102532

**Published:** 2019-10-21

**Authors:** Marzena Jezewska-Zychowicz, Jerzy Gębski, Marta Plichta, Dominika Guzek, Małgorzata Kosicka-Gębska

**Affiliations:** Department of Food Market and Consumer Research, Institute of Human Nutrition Sciences, Warsaw University of Life Sciences (SGGW-WULS), 159C Nowoursynowska Street, 02-787 Warsaw, Poland; marzena_jezewska_zychowicz@sggw.pl (M.J.-Z.); marta_plichta@sggw.pl (M.P.); dominika_guzek@sggw.pl (D.G.); malgorzata_kosicka_gebska@sggw.pl (M.K.-G.)

**Keywords:** overweight, obesity, diet, physical activity

## Abstract

Obesity is a serious problem for both the individual and society due to its health and economic consequences. Therefore, there is a need to focus on factors which explain this phenomenon and may be useful in preventing future occurrence. The aim of this study was to determine the lifestyle factors coexisting with increased body mass index (BMI ≥ 25 kg/m^2^) in Polish adults, including factors related to diet (dietary patterns—DPs; dietary restrictions; number of meals; frequency of snacking, eating out, and ordering home delivery meals), physical activity, and sociodemographic characteristics. A cross-sectional quantitative survey was carried out in 2016 amongst 972 Polish adults under the Life Style Study (LSS). To determine the factorscoexisting with BMI ≥ 25 kg/m^2^, the logistic regression model was developed. Women were less likely to be overweight or obese compared to men. The likelihood of BMI ≥ 25 kg/m^2^ increased with age by 4% in each subsequent year of life. Frequent consumption of fruits and vegetables, adhering to restrictions in quantity of food consumed and at least moderate physical activity during leisure time decreased the likelihood of BMI ≥ 25 kg/m^2^. More frequent consumption of meat and eating five or more meals a day increased the likelihood of BMI ≥ 25 kg/m^2^. Diet-related factors explained the developed model better than factors related to physical activity, however, age and gender were the factors most strongly correlated with BMI ≥ 25 kg/m^2^. Therefore, development of strategies to prevent and reduce overweight and obesity should focus on the demographic characteristics of the population, and then on teaching behaviors conducive for reducing the amount of food consumed, especially meat. However, physical activity in leisure time should also be included in the prevention of obesity.

## 1. Introduction

Nowadays, obesity has become a serious challenge because of its growth rate and negative health consequences. The occurrence of obesity has increased fourfold in the last 40 years [1]. In 2016, 26% of men and 25% of women in Poland were obese [2]. Because weight gainmay cause both non-communicable diseases (NCDs) [3,4] and some psychological and social problems [5], there is a need to focus on explaining the reasons for this phenomenon and searching for methods to prevent obesity [6]. It is emphasized that population- or community-level interventions for obesity should be intensively implemented on a larger social scale by promoting physical activity, reducing sedentary lifestyle, encouraging healthy diet and optimal sleep duration [7]. The modifications of these behavioral factors may cause a change in the individual’s lifestyle that will help maintain a healthy body weight.

It has been observed that an individual’s lifestyle can substantially contribute to increase in body weight [8,9]. Previous studies have shown that diet and physical activity are the most important risk factors for overweight and obesity [10,11,12,13]. So far, the relationship between an intake of specific food products, nutrients, or energy and the occurrence of overweight and obesity predominated in the studies [14,15,16]. Although, only a few studies have investigated the relationship between dietary patterns (DP) and body weight, they suggest that DP (clusters of eating behaviors) may be more relevant in explaining weight gain than any assessment of a single dietary component [14,15,16]. Simultaneously, recent studies have shown that other diet characteristics including time allocation for meals, restricting eating hours and regularity of food consumption may also affect the total energy intake and hence lead to the increase in body weight [17,18]. In addition, it has been shown that skipping breakfast [19], taking in the majority of total energy in the evening [20], eating away from home [21], and higher frequency of snacking [22] associate positively with overweight and obesity. Energy expenditure is in turn linked to physical activity. Low physical activity is often associated with sedentary behaviors [23,24,25], which highly reduces the expenditure of energy supplied from food. Also, being physically inactive and unhealthy dietary patterns, e.g. eating high-energy foods, increase the likelihood of accumulating body fat and increasing body weight [16,26].

Although research is being conducted intensively on the causes of obesity, there is still a deficiency in studies whereby the components of lifestyle are taken into account as a more complex set when diagnosing factors contributing to the risk of obesity. Thus, the aim of this study was to determine the lifestyle factors coexisting with increased body mass index (BMI ≥ 25 kg/m^2^) in Polish adults, including factors related to diet (dietary patterns—DPs; dietary restrictions; number of meals; and frequency of snacking, eating out, and ordering home delivery meals), physical activity, and sociodemographic characteristics. We assumed that: (1) the diet- related factors (i.e., dietary patterns; restrictions in food consumption; number of meals; and frequency of snacking, eating out, and ordering home delivery meals) correlated more stronger with having BMI ≥ 25 kg/m^2^ than physical activity (self-reported physical activity and sedentary behaviours); (2) the associations between specific diet-related factors and having BMI ≥ 25 kg/m^2^ are varied; (3) BMI is associated with socio-demographic characteristics.

## 2. Materials and Methods 

### 2.1. Study Design and Sample Collection

The study was approved by the Ethics Committee of the Faculty of Human Nutrition and Consumer Science, Warsaw University of Life Sciences, in Poland on the 27th of June, 2016 (Resolution No. 01/2016). Informed consent to participate in the study was collected from participants.

A cross-sectional quantitative survey was carried out to collect the data using the computer-assisted web interviewing (CAWI) technique. Adults aged 21–65 were recruited by a research agency from the panel of approximately 55,000 people. After sending an invitation to participate in the LifeStyle Study (LSS), 6910 people agreed to participate in the study. Quota selection using gender, age, place of residence, and region was used to ensure the representativeness of the Polish population. During the recruitment, five people did not meet the panel criteria (age above 65), 144 people stopped filling out the questionnaire during the interview, and 5746 people did not qualify after filling the quota. At the collection control stage, eight people were removed from the database because of errors showing the lack of credibility of their answers. The study sample consisted of 1007 participants. The results of LSS study showing the relationship between overweight/obesity and sleep duration [6], and the relationship of the dietary patterns with physical activity and sedentary behaviors have already been published [9].

To achieve the goal of the study, one more criterion of exclusion was used, namely, being underweight. Thirty-five participants were excluded from the analyses due to BMI lower than 18.5 kg/m^2^. The total sample consisted of 972 people. 

### 2.2. Diet-Related Factors

#### 2.2.1. Frequency of Food Eating

In order to assess the frequency of consumption of selected food groups, the Beliefs and Eating Habits Questionnaire (KomPAN) was used [7]. All participants were asked to record their habitual eating frequency within the last year according to the following food groups: wholemeal bread; wholemeal pasta and groats; fermented milk drinks; cheeses; cured meats and sausages; red meat; white meat; fried foods; fruits; vegetables; vegetable juices; fruit juices; fizzy drinks; meals or snacks such as burgers, pizza, chicken, fries; sweets and cakes; crisps and other salty snacks. The following frequency categories were used: 1—never; 2—less often than once a month, 3—from 1 to 3 times a month; 4—once a week; 5—several times a week; 6—once a day; 7—several times a day. 

#### 2.2.2. Dietary Restrictions

To collect data about dietary restrictions applied in the last 6 months, questions regarding 10 categories of restrictions were used. The restrictions considered in the study included food quantity, sugar and/or sweets, high-fat foods, fats, cereals and/or bread and/or potatoes, meats, fish, dairy products, raw vegetables, and raw fruit [27]. ‘Yes/no’ answers were used to record the application of particular restrictions.

#### 2.2.3. Other Eating Habits

Participants were asked to assess the frequency of eating outside the home, snacking and ordering home delivered meals within the last year.The following frequency categories were used: 1—never; 2—less often than once a month; 3—from 1 to 3 times a month; 4—once a week; 5—several times a week; 6—once a day; 7—several times a day. The number of meals per day was recorded in the questionnaire as well.

### 2.3. Physical Activity and Sedentary Behaviors

Self-reported physical activity was assessed separately for the physical activity during leisure and work/school time. This was recorded on a three-point scale: 1—‘low’; 2—‘moderate’; 3—‘high’ [7]. The description of the three-point scale was different for leisure and work/school time. ‘Low’ physical activity in leisure time was described as ‘sedentary lifestyle, watching TV, reading the press, books, light housework, taking a walk for 1–2 hours a week’; ‘moderate’ one as ‘walks, cycling, gymnastics, gardening or other light physical activity performed for 2–3 hours a week’, and ‘high’ one as ‘cycling, running, working on a plot or garden, and other sports activities requiring physical effort, taking up more than 3 hours a week’. The description of activity at work/school time was as follow: ‘low‘—‘over 70% of the time in a sitting position’; ‘moderate’—‘approximately 50% of the time in a sitting position and moving for about 50% of the time’; and ‘high’—‘being in motion for about 70% of the time or doing physical work associated with a lot of effort’ [7]. 

Sedentary behaviors included reading books and newspapers, watching TV and using the computer. Frequency of each sedentary behavior was recorded on a seven-point scale described as follows: 1—never; 2—less than once a month; 3—1–3 times a month; 4—once a week; 5—2–6 times/week; 6—once a day; to 7—more than once a day. 

### 2.4. Socio-Demographic Characteristics

Questions about such sociodemographic characteristics of the sample as gender, age, education, and place of residence were included in the questionnaire. In order to calculate Body Mass Index (BMI), participants were asked to self-report body weight and height. The BMI categories were identified according to International Obesity Task Force (IOTF) standards [28]. During the data analysis, the BMI categories below 18.5 kg/m^2^ were excluded from the sample and two categories of respondents were identified—i.e., those with normal weight (BMI between 18.5 kg/m^2^ and 24.99 kg/m^2^), and those who are overweight or obese (BMI ≥ 25 kg/m^2^).

### 2.5. Statistical Analysis

Descriptive statistics were performed. The chi-square test and Student’s *t*-test were used to compare variables, and *p* < 0.05 was considered significant. A factor analysis with principal component extraction method was conducted to derive dietary patterns based on the frequency of eating from 16 food groups [6]. In brief, the factors were rotated by an orthogonal (Varimax) transformation. Such criteria as components with an eigenvalue of 1, a scree plot test, and the interpretability of the components were used to determine the number of components. The total variance explained was 66.2%. Loadings equal to 0.50 or higher were used to identify the component of the dietary patterns. Five dietary patterns (principal components) were identified: ‘Fruit & vegetable’ DP (F&V), ‘Wholemeal food’ (WF); ‘Fast foods & sweets’ (FF&S); ‘Fruit & vegetable juices’; ‘Meat & meat products’ (M&MP) [6].

The tertile distribution was usedto divide the participants into three groups within each DP (bottom, middle, upper tertile). The upper tertile represents the greatest adherence, while the bottom tertile represents the lowest adherence to the DP. 

In order to estimate the chance of being overweight or obese (BMI ≥ 25 kg/m^2^), a regression model was developed, in which the dependent variable wasin BMI categoryand treated as the dichotomous variable (18.5 kg/m^2^ ≤ BMI < 25 kg/m^2^ vs BMI ≥ 25 kg/m^2^). The independent (explanatory) variables entered to the model were: DPs, dietary restrictions, frequency of eating outside the home, snacking and ordering home delivered meals, self-reported physical activityduring leisure time and work/school time, reading books and newspapers; watching TV; using the computer, and sociodemographic variables (gender, age, education, place of residence). Automatic selection of variables to the model with the stepwise method was applied. Only significant variables at the level of *p* < 0.05 have been used.

The occurrence of possible multicolinearity was assessed using the correlation coefficient between the variables used in the model. No high correlation coefficients were observed between the explanatory variables in the correlation matrix. The highest value of 0.194 was between age and ‘Fruit & vegetable’ DP. Next, we examined multicollinearity through the variance inflation factor and tolerance. The minimum observed value of ‘tolerance’ was 0.895, which confirmed a lack of colinearity. The maximum value of ‘variance inflation’ was 1.113, which did not confirm multicolinearity as well [29].

The pseudo-R^2^ model fit was used to assess the quality of the model. Its value is 0.271 (i.e., its maximum scaled value is 0.312). The hypothesis ‘H0: all the parameters β in the model are equal to 0’was rejected at every level of significance, both for the likelihood ratio, and the Wald statistics. The Hosmer and Lemeshow test was used to calculate the probabilities of all the observations divided into 10 groups [30]. Statistics χ2 analyzed the differences between the observed and expected value. The high *p*-value equal to 0.344 does not allow the rejection of the H0 hypothesis assuming the correspondence of the theoretical and empirical values.Therefore, it confirms that the model is well suited. The adequacy of the model was assessedusing a standard significance level of 0.05.

All analyses were performed using SAS 9.4. software (SAS Institute, Cary, NC, USA). 

## 3. Results

### 3.1. Sample Characteristics

The study sample consisted of 972 participants (499 women and 473 men) aged 21 to 65 years. Some details concerning socio-demographic characteristics of the study sample are displayed in Table 1. 

Normal weight (18.5 kg/m^2^ ≤ BMI < 25 kg/m^2^) had 484 study participants and 488 participants were overweight or obese (BMI ≥ 25 kg/m^2^). More men than women were overweight or obese. More than twice as many people with higher education had normal weight compared to those with secondary education. Among people with normal weight, the majority were people between the ages of 21-34, while among those with BMI ≥ 25 kg/m^2^, people aged 55–65 were the most numerous (Table 1).

### 3.2. Lifestyle Characteristics 

Nearly two-thirds (66.4%) of the sample declared obeying some restrictions in food consumption, 11.7% declared continuous restrictions, and 54.7%—occasional restrictions. Over two-fifths of the sample (42.8%) implemented restrictions in quantity of food consumed. Limitations of sugar and sweets were declared by 48.3% of people, animal and vegetable fats by 25.2% (Table 2).

Using the Internet on a daily basis was declared by 92.3% of people, 39.4% of respondents read books/press, and 69.1% watched TV. Snacking at least once a day was declared by 31.3% of people. Ordering meals delivered to the house at least 1–3 times a month was declared by 32.7% of people, while eating outside of the home at least once a week was declared by 23.2% of people. Almost half of the study sample (48.8%) described their physical activity at work as high or moderate, while such level of physical activity in leisure time was reported by 64.3% of people (Table 2). 

More people with BMI ≥ 25 kg/m^2^ compared to others declared obeying restrictions in quantity of food consumed and in sugar and sweets, while more people with BMI < 25 kg/m^2^ used the Internet and watched TV at least once a day. More people with BMI ≥ 25 kg/m^2^ declaredlow physical activity than others and more people with BMI < 25 kg/m^2^ reported their high physical activity in leisure time (Table 2). 

### 3.3. Logistic Regression Model 

Explanatory variables of the regression model are presented in Table 3. Only two DPs, restriction in quantity of food consumed and physical activity in leisure time as lifestyle variables significantly correlated with being overweight or obese (BMI < 25 kg/m^2^) at the level *p* < 0.05. However, gender and age had (in the model) the strongest effect in explaining underweight or obesity. 

People in the middle and upper tertiles of ‘Meat and meat products’ DP (OR: 1.51; 95% CI: 1.08–2.13, and OR: 1.71; 95% CI: 1.21–2.42, respectively) were more likely to be overweight or obese compared to those at the bottom tertile of the DP (low frequency of consumption). In the upper tertile of ‘Fruit & vegetable’ DP, the risk of overweight or obesity decreased compared to the bottom tertile (OR: 0.65; 95% CI: 0.45–0.93). The risk of BMI ≥ 25 kg/m^2^ in people who did not obey any restrictions regarding the quantity of consumed food was twice as high compared to people adhering to such restrictions (OR: 2.00; 95% CI: 1.49–2.68). People consuming five or more meals a day were more likely to have BMI ≥ 25 kg/m^2^ (OR: 1.78; 95% CI: 1.21–2.64) compared to those eating three or less meals a day (Table 4). Such DPs as ‘Fast foods & sweets’, ‘Wholemeal food’, and ‘Fruit and vegetable juices’, but also restrictions in consumption of various food products, snacking, eating outside the home, ordering home delivered meals did not significantly explain being overweight and obese.

Both people assessing their leisure activity as moderate (OR: 0.71; 95% CI: 0.52–0.96) and high (OR: 0.49; 95% CI: 0.32–0.74) were less likely to be overweight and obese compared to those who declared low physical activity (Table 4). Self-reported physical activity at work and all tested sedentary behaviors did not significantly explain having BMI ≥ 25 kg/m^2^.

Women were less likely to have BMI ≥ 25 kg/m^2^ compared to men (OR: 0.35; 95% CI: 0.26–0.46). Higher chance of BMI ≥ 25 kg/m^2^ was observed in people with secondary education (OR: 1.59; 95% CI: 1.18–2.13) compared to those with higher education. The chance of BMI ≥ 25 kg/m^2^ increased with age. Each subsequent year of life increased the risk of overweight and obesity by 4% (OR: 1.04; 95% CI: 1.03–1.05) (Table 4).

## 4. Discussion

Obesity is a global problem, with differences in its occurrence after taking into account sociodemographic characteristics [31,32]. In our study, age and gender best explained overweight and obesity among all the variables entered in the developed model. There are more obese women than men in the world [31], but the opposite is observed in developed countries, as well as in Poland [2]. In the study sample, there were also more menwho had BMI ≥ 25 kg/m^2^. The chance of overweight and obesity was almost three times lower in women compared to men. This may be due to the greater attention paid by women to body weight and health, which usually has a positive effect on their lifestyle [33]. Nevertheless, more women than men experienced various eating disorders, which can be associated with a greater desire to maintain low body weight, especially in young women [34]. Among postmenopausal women, there was an increased risk of weight gain [35]. The increased risk of BMI ≥ 25 kg/m^2^ with age has also been confirmed in the study sample. The importance of sociodemographic variables (age and gender) in explaining obesity still require further research.

Similarly to other studies [20,36,37], our findings have shown a relationship between having BMI ≥ 25 kg/m^2^ and higher frequency of eating meat (‘Meat & meat products’ DP). It can therefore be assumed that the prevention of obesity requires a reduction in meat consumption, both its quantity and frequency of consumption. However, it might be difficult as meat is one of the most popular food products in many countries [38] and is still perceived as healthy food [39]. Such changes are particularly difficult in countries where meat is a cultural symbol [40]. In Poland, meat consumption is still a symbol of prestige and wealth, which is a serious barrier to its limitation. Limiting the energy from meat consumption by choosing lean meat could help reduce weight gain. However, this effect is difficult to achieve, because oftentimes the energy of a meal cannot be determined by the meat itself, but by the way it is prepared and the other high-energy components of the meal. Thus, further research or other methods of analysis (e.g., cluster analysis) are needed to identify homogeneous groups in terms of dietary patterns to confirm the assumption that not the meat itself but the way it is prepared and incorporated into a meal has an effect on weight gain.

Other controversies and uncertainties about links between meat consumption and obesity still remain [41], especially the ones relating to the consumption of red meat and other processed meats [36,41]. In our study, the frequency of eating red meat, white meat and cured meats and sausages correlated positively within the M&MP pattern, this prevented us from examining the importance of the frequency of red meat consumption in predicting obesity.

In our study, we did not find an association between FF&S and being overweight or obese as confirmed in some other studies [13,42]. However, most of the previous studies indicated that BMI ≥ 25 kg/m^2^ is associated with a high intake of fast foods, and high consumption of sugar-sweetened beverages [43,44]. The inconsistency in results may in part be explained by the difference in the method of measuring fast-food and sweets consumption. In addition, the measurement error associated with using self-reported body weight and height to calculate BMI may have contributed to this non-compliance because overweight and obese people might under-report their consumption of fast food and sweets [45]. Thus, studies focusing on explaining the relationship of fast food consumption and weight should be continued, especially with the use of different measurement, i.e., both habitual frequency of eating and fast food and sweets intake across a long period of time due to possible differences between days. The use of dietary restrictions in limiting both the amount of food and the consumption of some products could also contribute to a lower frequency of eating fast-food, thereby reducing energy from the diet, especially in overweight and obese people. Our findings have shown that the risk of BMI ≥ 25 kg/m^2^ was higher among people who did not obey any restrictions regarding the amount of food consumed. At the same time, more people with BMI ≥ 25 kg/m^2^ applied restrictions in the quantity of consumed food and also in sugar and sweets. Thus, the use of dietary restrictions, especially for high-energy foods, may partly explain the lack of a positive relationship between ‘Fast food & sweets’ DP and BMI ≥ 25 kg/m^2^.

We have not observed any association between ‘Wholemeal food’DP and weight status. In previous studies, intake of dietary fiber from grains was associated with significantly lower body weight [15,46]. In the context of current knowledge, the lack of a relationship between the consumption of whole grains and body weight in the study sample requires explanation. Still, low consumption of whole-grain food in Poland can be considered the main reason for not observing its positive impact. This is mainly due to its higher prices and low availability of this food, especially in rural areas and smaller cities.

Similarly to other studies [47], we found that people following a diet rich in fruits and vegetables (F&V DP) were less likely to be overweight and obese. However, the relationship between ‘Fruit & vegetable juices’ DP and weight status was not observed. As with whole grains, this can be explained by the low juice intake in Polish adults. Increasing the consumption of juices in adults, especially fruit juices, in order to prevent obesity should not be recommended due to their relatively high energy intake. Thus, our findings justify recommending increased fruit and vegetable intake for preventing and/or treating obesity [46]. However, such recommendation without explicitly combining this approach with efforts to reduce intake of other energy sources are unwarranted [43]. 

Apart from DPs, the restrictions regarding quantity of food consumed and consumption of five or more meals a day have also shown associations with being overweight and obese. Findings from previous research suggested that dietary restrictions can be linked with both higher and lower BMI. Higher BMI may reflect repeated unsuccessful attempts at weight control [44,48]. However, other studies have shown relationship of no-restraint or low-restraint with overweight and obesity [37,49]. Our findings are in line with the latter. A chance of having BMI ≥ 25 kg/m^2^ in people who did not apply food restrictions was twice as high as in people with dietary restrictions. Moreover, some studies reported a positive relationship of low frequency of eating with obesity [50,51] which was not reflected in our results. However, there are also studies showing lack of relationship of BMI with home-cooked meals [52] or with more frequent meals similarly as in our study [53]. The results regarding association between the dietary restrictions, the number of meals consumed and body weight requires further research. There is also a need to determine the size of consumed meals, allowing a more thorough interpretation of the relationship between variables.

Similarly to some studies [54,55], our findings confirmed that being overweight or obese did not correlate with eating out, as well as with snacking [56]. However, positive correlation of frequent fast-food consumption away from home with BMI was established as well [21,57]. It was also found that frequent restaurant visits associate positively with the BMI [57], which was in contrast to the results of other studies [58,59]. The lack of this relationship in the study sample may result from the lower popularity of using catering services in Poland than in other countries [60], and especially with regard to fast food restaurants [61]. The differences found in the results of previous studies on the importance of various diet-related factors in contributing to overweight and obesity may be due to cultural background. Therefore, to create strategiesaimed to prevent and reduce overweight and obesity, there is need to conduct research in the population to which these strategies are addressed.

Regular physical activity protects against long-term weight gain [62]. Similarly to the study of Abu-Omar & Rütten [63], we observed the relationship between BMI and leisure-time physical activity, but not with work/school physical activity. Absence of sedentary behaviors in explaining the BMI ≥ 25 kg/m^2^ in the model is surprising and inconsistent with the results of previous studies. Results of previous studies showed that watching TV and using the computer caused weight gain in adults [64]. Sedentary behaviors including watching TV were positively related to a high consumption of sweetened beverages, ready to eat products, sweet food, snacks, fast foods, and alcohol [14]. However, these foods did not correlate with BMI ≥ 25 kg/m^2^ in our sample. The lack of relationship between the sedentary behaviors and weight status may result from the under-estimated frequency when using self-reported measures. 

The strength of our results is a relatively large representative sample of Polish population in terms of the region of residence and gender. Although, our findings are specific to Polish population and should not be generalized to the population of other cultural background, the observations could be of potential use in designing interventions in some countries, that are somehow similar to Poland in terms of economic transformation or with a short history of nutritional education in public schools. We believe that including both lifestyle components in the analysis is of great importance for health, i.e., diet and physical activity brought a wider perspective on factors explaining overweight and obesity.

Nevertheless, there are several limitations regarding the study. One of them relates to the potential biases that may occur when self-reported data are analyzed. Regarding self-reported weight and height, men typically overestimated height and women underestimated weight in self-reported health measures [45,65]. Moreover, measures of adiposity such as waist circumference (WC) or the waist–hip-ratio (WHR) may be better indicators of obesity risk and mortality than BMI, for example, among the elderly [66]. Self-reported indicators of lifestyle can be considered not quite satisfying, however, using other measurement indicators confirm the results from the analysis of self-reported data [67]. Secondly, it could be argued that the use of FFQs led to overestimation of food consumption [68]. However, FFQ was chosen because we aimed to see predominantly ‘healthy’ and ‘unhealthy’ dietary patterns, rather than exact amount of foods. Additionally, this cross-sectional study design does not provide an opportunity to find a causal relationship between socioeconomic and lifestyle variables. Finally, the approach of dietary patterns did not consider vegetables and fruits separately in search of a connection between their consumption and increased body weight, which was suggested by other researchers [60,69].

## 5. Conclusions

We found the associations between having BMI ≥ 25 kg/m^2^ and‘Meat & meat products’ DP; ‘Fruit & vegetable’ DP; restrictions in quantity of food consumed; eating five or more meals a day; self-reported physical activity at leisure time; and such sociodemographic characteristics as gender, age, and education. Diet-related factors explained the model better than factors related to physical activity, therefore, their inclusion in interventions and educational programs which focused on prevention and reduction of overweight and obesity may be more effective. Association between restrictions in quantity of food consumed and BMI has shown that the ability to control the frequency and uantity of food consumed seems to be animportantfactor in preventing weight gain. This applies to both food commonly perceived as healthy (fruit and vegetable) and to foods perceived as less healthy (meat). Other lifestyle components both concerning diet (‘Wholemeal food’, ‘Fast foods & sweets’, and ‘Fruit & vegetable juices’ DPs; other dietary restrictions; and frequency of eating out and snacking, physical activity (at work), sedentary behaviors) did not show relationships with weight status in the study sample. Age and gender were the factors which best explain overweight and obesity in the developed model. Therefore, development of strategies to prevent and reduce overweight and obesity should be focused on the demographic characteristics of the population, and then on teaching behaviors conducive to reducing the amount of food consumed, especially meat. However, physical activity in leisure time should also be included in the prevention of obesity.

## Figures and Tables

**Table 1:** Characteristics of the study sample.

Variables		Total Sample		18.5 kg/m^2^ ≤ BMI < 25 kg/m^2^		BMI ≥ 25 kg/m^2^		*p*
		N = 972	100%	N = 484	100%	N = 488	100%	
Gender *	Female	499	51.3	305	63.0	194	39.8	<0.0001
	Male	473	48.7	179	37.0	294	60.2	
Education *	Secondary and lower	388	39.9	156	32.2	232	47.5	<0.0001
	Higher	584	60.1	328	67.8	256	52.5	
Place of residence	City ≤ 50 000 residents	195	20.1	88	18.2	107	21.9	0.3004
	City > 50 000 residents	521	53.6	269	55.6	252	51.7	
	Rural area	256	26.3	127	26.2	129	26.4	
Age *	21–34 years	346	35.6	227	46.9	119	24.4	<0.0001
	35–44 years	228	23.5	116	24.0	112	23.0	
	45–54 years	131	13.5	49	10.1	82	16.8	
	55–65 years	267	27.4	92	19.0	175	35.8	
Age (years) **	Mean; standard deviation	42.1; 13.1		38.6; 12.5		45.6; 12.8		<0.0001
Height (cm) **	Mean; standard deviation	171.7; 9.1		170.7; 8.8		172.7; 9.4		0.0007
Weight (kg) **	Mean; standard deviation	75.4; 15.4		65.0; 9.1		85.7; 13.4		<0.0001
BMI (kg/m^2^) **	Mean; standard deviation	25.5; 4.3		22.3; 1.8		28.7; 3.5		<0.0001

N—Number of participants; * Significance between BMI groups (Chi-square test); ** Significance between BMI groups (*t*-test).

**Table 2:** Lifestyle characteristics of the study sample.

Variables	Total Sample		18.5 kg/m^2^ ≤ BMI < 25 kg/m^2^		BMI ≥ 25 kg/m^2^		*p*
	N	%	N	%	N	%	
Total sample	972	100.0	484	100.0	488	100.0	
Restriction in quantity of consumed food	416	42.8	181	37.4	235	48.2	0.0007
Restriction in sugar and sweets	469	48.3	216	44.6	253	51.8	0.0244
Restriction in products like bread, potato, rice	129	13.3	57	11.8	72	14.8	0.1713
Restriction in meat and meat products	119	12.2	68	14.0	51	10.5	0.0870
Restriction in dairy products	67	6.9	38	7.9	29	5.9	0.2402
Restriction in animal and vegetable fats	245	25.2	120	24.8	125	25.6	0.7681
Restriction in meat and cured meats	212	21.8	95	19.6	117	24.0	0.1008
Restriction in fish	17	1.7	11	2.3	6	1.2	0.2148
Restriction in food containing gluten and/or lactose	54	5.6	24	5.0	30	6.1	0.4185
Number of meals a day:							
3 meals or less	405	41.7	201	41.5	204	41.8	
4 meals	370	38.1	190	39.3	180	36.9	0.6407
5 meals and more	197	20.3	93	19.2	104	21.3	
Snacking at least once a day	304	31.3	155	32.0	149	30.5	0.9482
Frequency of eating outside the home at least once a week	225	23.2	101	20.9	124	25.4	0.0688
Frequency of ordering meals at least 1–3 times a month	318	32.7	147	30.4	171	35.0	0.0836
Watching TV at least once a day	672	69.1	365	75.4	307	62.9	0.0001
Using the Internet at least once a day	897	92.3	459	94.8	438	89.8	0.0375
Reading books, newspapers at least once a day	383	39.4	190	39.3	193	39.5	0.7639
Sleep duration at weekdays at least 7 hours	684	70.4	331	68.4	353	72.3	0.1111
Sleep duration at weekend days at least 7 hours	843	86.7	424	87.6	419	85.9	0.6866
Physical activity during work/school time							
Low	498	51.2	239	49.4	259	53.1	
Moderate	381	39.2	195	40.3	186	38.1	0.4662
High	93	9.6	50	10.3	43	8.8	
Physical activity during leisure time							
Low	347	35.7	152	31.4	195	40.0	
Moderate	461	47.4	236	48.8	225	46.1	0.0056
High	164	16.9	96	19.8	68	13.9	

N—Number of participants; Significance between BMI groups (Chi-square test).

**Table 3:** Variables of the regression model.

Categories	Model	
	χ^2^ Wald’s	*p*
Gender	49.63	<0.0001
Age	46.69	<0.0001
Restriction in quantity of food consumed	21.58	<0.0001
Physical activity at leisure time	11.89	0.0030
‘Meat & meat products’ DP	10.11	0.0060
Education	9.25	0.0098
Numbers of meals a day	8.55	0.0140
‘Fruit & vegetable’ DP	6.20	0.0450

**Table 4:** Overweight and obesity (BMI ≥ 25 kg/m^2^) odds ratios (OR; 95% CI) in the study sample.

Parameter	Level of Variable	Model				
		β	OR	95% Cl		*p*
**Intercept**		−1.604				<0.001
Gender	Male (ref)	0	1	-	-	
	Female	−1.063	0.35	0.26	0.46	<0.001
Age (years)		0.040	1.04	1.03	1.05	<0.001
Restriction in quantity of consumed food	Yes (ref.)	0	1	-	-	
	No	0.694	2.00	1.49	2.68	<0.001
Physical activity during leisure time	Low (ref.)	0	1	-	-	
	Moderate	−0.350	0.71	0.52	0.96	0.027
	High	−0.724	0.49	0.32	0.74	0.001
‘Meat & meat products’ DP	Bottom tertile (ref.)	0	1	-	-	
	Middle tertile	0.415	1.51	1.08	2.13	0.017
	Upper tertile	0.535	1.71	1.21	2.42	0.003
Education	Higher (ref.)	0	1	-	-	
	Secondary	0.461	1.59	1.18	2.13	0.002
	Lower than secondary	0.146	1.16	0.25	5.40	0.852
Numbers of meals a day	3 meals and less (ref.)	0	1	-	-	
	4 meals	0.132	1.14	0.83	1.57	0.414
	5 meals and more	0.578	1.78	1.21	2.64	0.004
‘Fruit & vegetable’ DP	Bottom tertile (ref.)	0	1	-	-	
	Middle tertile	−0.102	0.90	0.64	1.27	0.557
	Upper tertile	−0.439	0.65	0.45	0.93	0.017

OR—point estimate; (95% Cl)—95% Wald Confidence Limits; ref.—reference group.
